# Titanium vs PEO Surface-Modified Magnesium Plate Fixation in a Mandible Bone Healing Model in Sheep

**DOI:** 10.1021/acsbiomaterials.4c00602

**Published:** 2024-07-29

**Authors:** Marta Turostowski, Carsten Rendenbach, Paulina Herzog, Agnes Ellinghaus, Ana Prates Soares, Max Heiland, Georg N. Duda, Katharina Schmidt-Bleek, Heilwig Fischer

**Affiliations:** † Department of Oral and Maxillofacial Surgery, Charité − Universitätsmedizin Berlin, Corporate Member of the Freie Universität Berlin, Humboldt-Universität zu Berlin and Berlin Institute of Health, Augustenburger Platz 1, Berlin 13353, Germany; ‡ Julius Wolff Institute, Berlin Institute of Health at Charité − Universitätsmedizin Berlin, Augustenburger Platz 1, Berlin 13353, Germany; § Center for Musculoskeletal Surgery, Charité − Universitätsmedizin Berlin, Corporate Member of the Freie Universität Berlin, Humboldt-Universität zu Berlin and Berlin Institute of Health, Augustenburger Platz 1, Berlin 13353, Germany; ∥ BIH Charité Clinician Scientist Program, Berlin Institute of Health at Charité − Universitätsmedizin Berlin, BIH Biomedical Innovation Academy, Charitéplatz 1 ,Berlin 10117, Germany

**Keywords:** magnesium, PEO, mandible fracture, miniplates, sheep

## Abstract

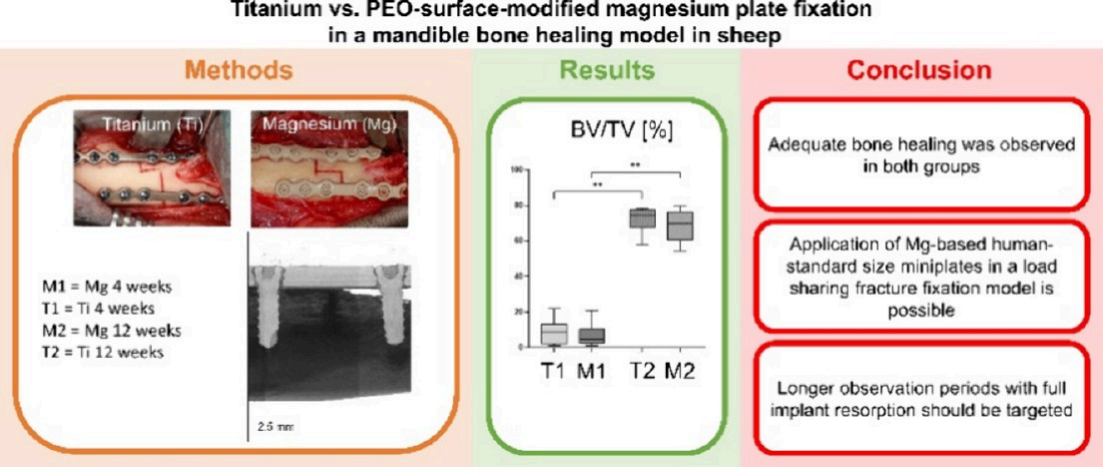

Titanium plates are the current gold standard for fracture fixation of the mandible. Magnesium alloys such as WE43 are suitable biodegradable alternatives due to their high biocompatibility and elasticity modulus close to those of cortical bone. By surface modification, the reagibility of magnesium and thus hydrogen gas accumulation per time are further reduced, bringing plate fixation with magnesium closer to clinical application. This study aimed to compare bone healing in a monocortical mandibular fracture model in sheep with a human-standard size, magnesium-based, plasma electrolytic-oxidation (PEO) surface modified miniplate fixation system following 4 and 12 weeks. Bone healing was analyzed using micro-computed tomography and histological analysis with Movat’s pentachrome and Giemsa staining. For evaluation of the tissue’s osteogenic activity, polychrome fluorescent labeling was performed, and vascularization was analyzed using immunohistochemical staining for alpha-smooth muscle actin. Bone density and bone mineralization did not differ significantly between titanium and magnesium (BV/TV: T1: 8.74 ± 2.30%, M1: 6.83 ± 2.89%, *p* = 0.589 and T2: 71.99 ± 3.13%, M2: 68.58 ± 3.74%, *p* = 0.394; MinB: T1: 26.16 ± 9.21%, M1: 22.15 ± 7.99%, *p* = 0.818 and T2: 77.56 ± 3.61%, M2: 79.06 ± 4.46%, *p* = 0.699). After 12 weeks, minor differences were observed regarding bone microstructure, osteogenic activity, and vascularization. There was significance with regard to bone microstructure (TrTh: T2: 0.08 ± 0.01 mm, M2: 0.06 ± 0.01 mm; *p* = 0.041). Nevertheless, these differences did not interfere with bone healing. In this study, adequate bone healing was observed in both groups. Only after 12 weeks were some differences detected with larger trabecular spacing and more vessel density in magnesium vs titanium plates. However, a longer observational time with full resorption of the implants should be targeted in future investigations.

## 1 Introduction

In displaced fractures of the mandible, open reduction and internal fixation with titanium plates and screws are the current gold standard.^1,2^ Despite good biocompatibility and sufficient biomechanical features to ensure bone healing, complications following implantation of nondegradable titanium occur, and plate removal is often necessary.^3−8^ The most frequently reported reasons for plate removal are infections, extrusion, facial deformity, and pain.^7,8^ Additionally, titanium causes imaging artifacts.^9^ As the e-modulus of titanium and bone is substantially different, its implantation entails the risk of stress shielding.^2,10^ Magnesium as a bioresorbable material affords several advantages, such as a reduced risk of stress shielding due to an elastic modulus closer to cortical bone, while radiological imaging is less compromised.^2,10,11^ Moreover, magnesium has beneficial effects on bone formation and may thus enhance the clinical outcome of surgical procedures.^2,12,13^ Previous in silico, ex vivo, and in vivo examinations have shown that the magnesium-based miniplates have similar biomechanical characteristics to titanium-based miniplates.^14−17^ The application of magnesium-based implants is concomitant with side effects of its corrosion. When exceeding the local resorption rate, the (hydrogen) gas formation may interfere with the healing process.^18−20^ To control the corrosion rate, a wide range of surface modifications and coatings were examined in previous studies.^19,21^ PEO markedly reduces the corrosion rate of magnesium-based implants in vivo.^22−24^ For certain indications, CE-certified WE43 cannulated screws are already in clinical use and demonstrate satisfactory results comparable to fixation with titanium screws.^25−29^ However, to the best of our knowledge, no magnesium-based plate fixation system for fracture treatment or orthognathic or reconstructive surgery is available. Despite there being sufficient data on the impact of WE43 with and without PEO surface modification on the degradation rate and effects on surrounding bone and soft tissues, studies on the effect of WE43 with PEO surface modification on bone healing are largely absent. This study aimed to investigate bone healing in the mandible under fixation with standard titanium miniplates versus fixation with biodegradable PEO surface modified WE43 magnesium miniplates in a large animal model.

## 2 Material and Methods

**Figure 1:**
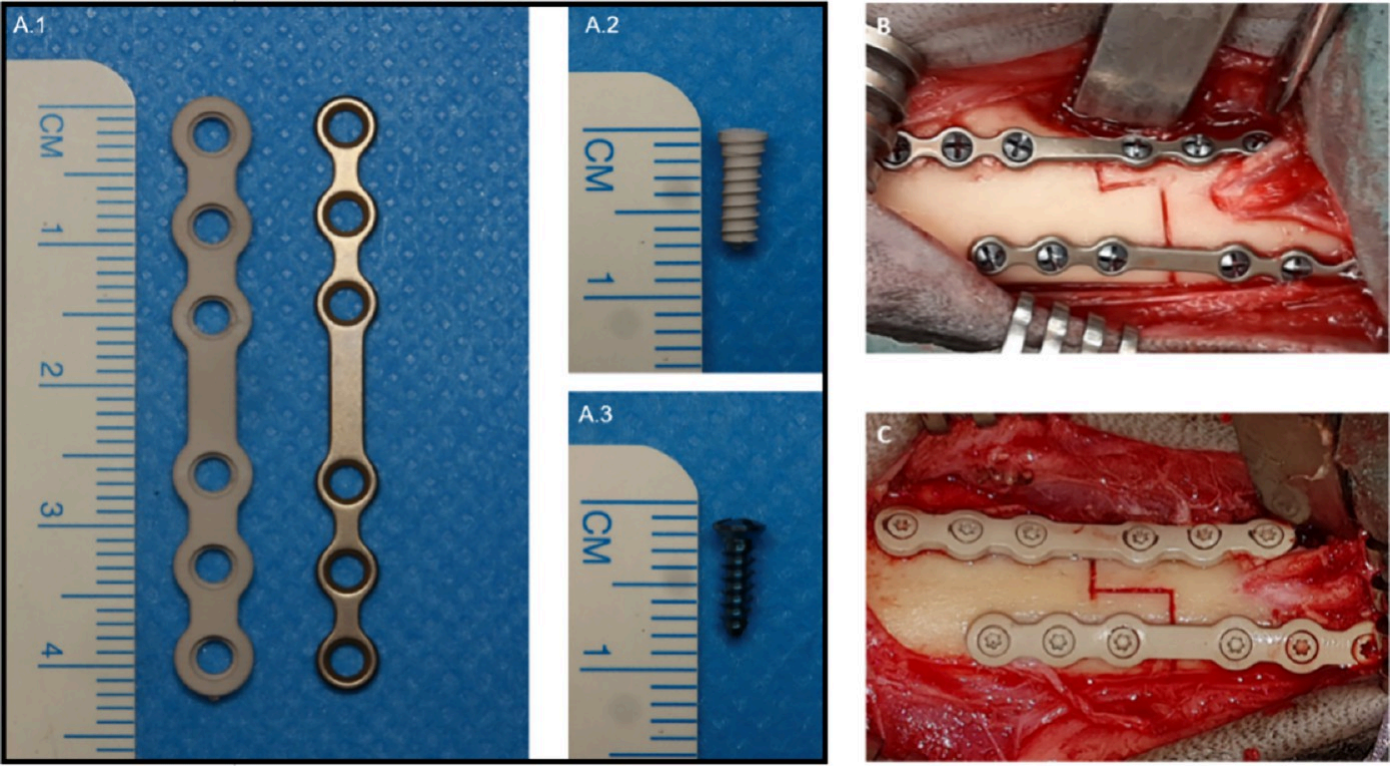
Test samples. (A.1) Magnesium plate (left) and titanium plate (right) prior to surgery. (A.2) Magnesium screw prior to surgery. (A.3) Titanium screw prior to surgery. (B) Intraoperative picture of titanium implants in the mandible. (C) Intraoperative picture of magnesium implants in the mandible.

**Figure 2:**
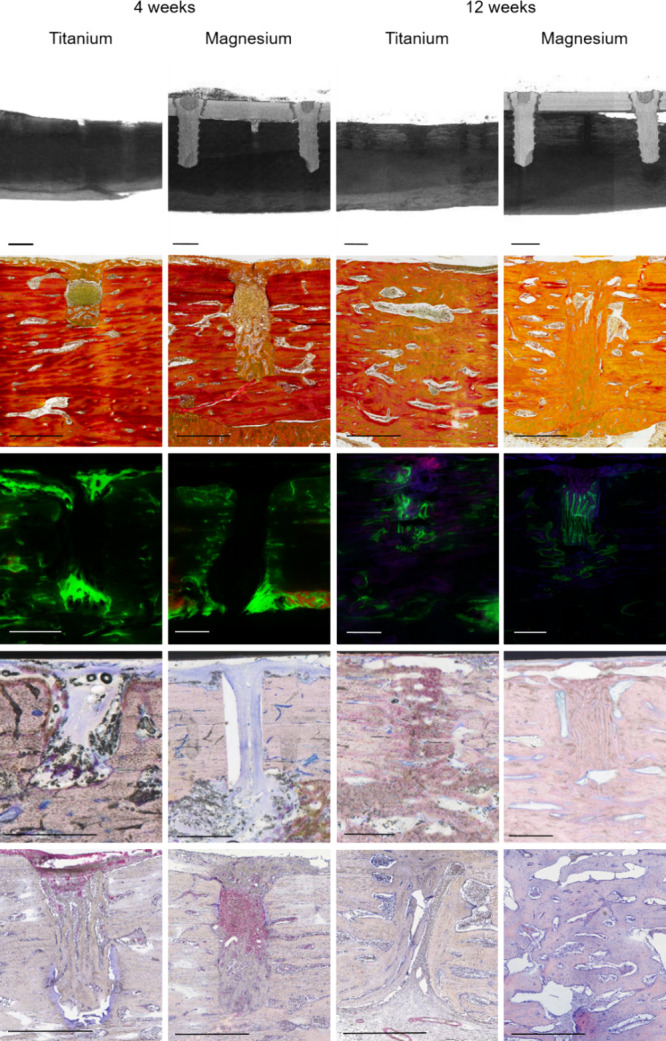
Overview of methodology. First row: reconstruction of micro-computed tomography for bone density and microstructure analysis. Scale bar: 2.5 mm. Second row: Movat’s pentachrome staining for quantification of newly mineralized bone and tissue composition. Scale bar: 1 mm. Third row: polychrome fluorescent labeling for quantification of osteogenic activity. Scale bar: 0.5 mm. Fourth row: Giemsa staining for analysis of tissue reaction surrounding the implant material. Scale bar: 1 mm. Fifth row: immunohistochemical staining for α-smooth muscle-actin to quantify the vascularization. Scale bar: 1 mm.

Bone formation was analyzed in a partial weight-bearing monocortical osteosynthesis model in sheep mandibles following 4 and 12 weeks. The noninferiority of the magnesium-based plates was analyzed radiologically and histologically to determine comparability to the standard fixation after the early bone healing phase and during the remodeling stage to further understand possible translatory approaches. Figures 1 and 2 illustrate an overview of the fixation material, surgical procedure, and histological and radiological methodology.

### 2.1 Test Samples

For the magnesium implants, a Mg-Y-RE-Zr alloy WE43MEO (Meotec GmbH, Aachen, Germany) was processed into six-hole miniplates with corresponding locking screws. The elemental fraction split out as follows: 1.4–4.2% Y; 2.5–3.5% Nd; and <1% Zn, Zr, Cu, Fe, Ni, Mn, and Al. Titanium miniplates representing the clinical gold standard were manufactured from pure titanium. Titanium screws were manufactured from Ti-6Al-4 V alloy. The manufacturing process included milling or extrusion and Swiss turning to obtain six-hole miniplates or locking screws, respectively. The present study compared 1.75 mm WE43MEO/PEO miniplates and 2.3 × 7 mm MaxDrive locking, self-tapping screws with 1.0 titanium miniplates and 2.3 × 7 mm MaxDrive locking, self-tapping screws. Regarding the micro design of the screws, the magnesium-based screws had a 0.1 mm wider core diameter. Plasma-electrolytic oxidation was performed as a surface modification for the WE43-based miniplates and screws. The manufacture of all implants used in this study was performed by the KLS Martin Group (Gebrüder Martin GmbH Co. KG, Tuttlingen, Germany). For detailed information on the PEO surface modifications, please refer to an earlier work from our team (Rendenbach et al., 2021).^38^

### 2.2 Animal Model

Ethical approval was obtained prior to this study (LaGeSo Berlin). The animal experiments were performed in accordance with the German Federal Animal Welfare Law and the guidelines for the care and use of laboratory animals and EU Directive 2010/63/EU for animal experiments.^30^ During the study, skeletally mature MerinoMix sheep were housed in groups under continuous veterinarian care. To guarantee the health and well-being of the animals, a veterinarian examination was carried out prior to inclusion into and throughout the study by laboratory animal veterinarians. Both the food supply and water were provided ad libitum in a humidity- and temperature-controlled environment. In total, 24 animals (24 females) with an average body weight of 66.8 kg (±13.5 kg) were included in the experiment. Following familiarization and surgery, the animals were observed for 4 and 12 weeks. Animals were randomly assigned to the titanium or magnesium plate groups; each group had *n* = 6 animals. T refers to the titanium groups; T1, the 4 week group; and T2, the 12 week group. The magnesium groups are referred to as M1/M2 accordingly. The subdivision into the four different groups occurred randomly. This study is reported according to the ARRIVE Guidelines for reporting animal research.^31^

### 2.3 Surgical Procedure

For sedation purposes, 10–15 mg/kg of BW thiopental-natrium was applied intravenously. Following intubation, the animals were anesthetized using 1.8–2.0 vol % isoflurane (CP-Pharma, Burgdorf, Germany). A continuous application of fentanyl (PanPharma, Trittau, Germany) over another intravenous catheter in the ear vein provided analgesia. For the perioperative antibiotic treatment, 3 g of ampicillin/sulbactam (2 g/1 g) (Dr. Friedrich Ebert Arzneimittel GmbH, Ursensollen, Germany) was applied as a single shot intravenously. A total of 1 L of Sterofundin and half a liter of balanced electrolyte solution (Sterofundin, Braun, Melsungen, Germany) were submitted during the procedure. The animals were placed on the operating table in the left lateral position. The operation was conducted under the aseptic conditions.

Following skin disinfection and sterile covering, a longitudinal skin incision of approximately 7 cm was placed over the right rostral part of the mandible body. Following blunt preparation of the soft tissue, the periosteum was incised and mobilized to directly access the mandible. An incomplete z-shaped monocortical 0.6 mm wide osteotomy was placed in the toothless area of the diastema between the corner incisor and the first premolar using a Piezotome saw (Piezosurgery medical, Mectron S.p.A., Carasco, Italy). Following the drilling of six 1.6 mm wide drilling holes, each vertical part of the z-shaped osteotomy was fixated using one mini plate with six screws. The periosteum was adapted, and a multilayered wound closure was performed. To prevent infection, an aluminum-based liquid bandage (CP-Pharma, Burgdorf, Germany) was extensively sprayed over the operation area. Meloxicam (0.5 mg/kg BW (body weight); Melosolute 20 mg/mL, CP-Pharma, Burgdorf Germany) was administered for at least 5 days and 15 mg/kg BW amoxicillin (Duphamox LA, Zoetis Deutschland GmbH, Berlin, Germany) was administered for 7 days to reduce postoperative pain and prevent infection, respectively.

### 2.4 Postoperative Interventions

To perform a polychrome fluorescent labeling, two or three different fluorescent substances were injected subcutaneously into the animals of the 4 and 12 week groups, respectively. In the 4 week group, 30 mg/kg BW alizarin red (alizarin-3-methyliminodiacetic acid; Sigma-Aldrich, Saint Louis, MO, USA) was injected after 7 days and 10 mg/kg BW calcein (Sigma-Aldrich, Saint Louis, MO, USA) after 21 days. In the 12 week group, 10 mg/kg BW calcein (Sigma-Aldrich, Saint Louis, MO, USA) was injected after 35 days, 30 mg/kg BW alizarin (alizarin-3-methyliminodiacetic acid, Sigma-Aldrich, Saint Louis, MO, USA) after 56 days, and 90 mg/kg BW xylenol (xylenol orange tetrasodium salt, Saint Louis, MO, USA) after 77 days. The sequences of the polychrome fluorescent labeling can be found in Section 3.5.

### 2.5 Postoperative Radiological Examination and Sacrifice

Radiological examination with X-rays was conducted to monitor early plate failure and dislocation. Following euthanasia, the fragment of the mandible representing the osteotomy area was extracted with an oscillating saw (Oscillating Saw 518.01, Depuy Synthes, West Chester, PA, USA) while maintaining a safe distance of 1 cm from the rostral and caudal end points of the miniplates. Using a Piezotome saw, the tissue between the two plates was divided. The first plate used for radiological and histological analysis in paraffin embedding was fixated in a 4% paraformaldehyde solution (Avantor Performance Materials, Gliwice, Poland), and the other plate used for fluorescence and histological analysis in PMMA embedding was fixated in a 10% formaldehyde solution (Avantor Performance Materials, Gliwice, Poland).

### 2.6 Laboratory Micro-CT and Volume Analysis

After the formaldehyde was washed out (see Section 2.7), a laboratory micro-CT with 8.01 μm voxel size, 360° scan in 0.3° steps, 70 kW, and 114 A (SkyScan N.v., Aartselaar, Belgium) was performed. Volume reconstructions were performed using the software provided by the system.

**Figure 3:**
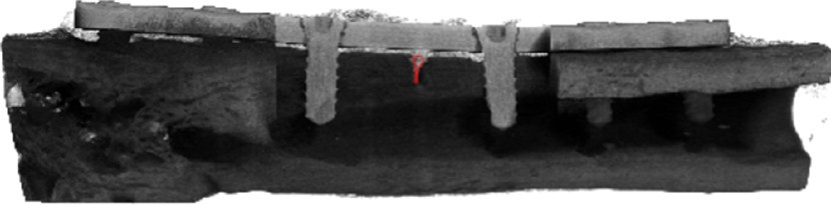
3D reconstruction of micro-computed tomography scan of a 4 week healed mandible with magnesium plate and screws. Red circle showing area of VOI placement. Scale bar: 2.5 mm.

The images were then imported to ImageJ (ImageJ 1.53o, National Institutes of Health, Rockville, USA). A self-implemented macro was used for both calibrations to mg HA/ccm with the help of a scanned phantom (HA Calibration Phantom, Scanco Medical AG, Brüttiselen, Switzerland) and for volume analysis. The histograms of all scans were evaluated to specify thresholds for quantifications. It was possible to identify a threshold of 835 mg of HA/ccm to separate bone and implant material from the surrounding tissue and air. A volume of interest (VOI) was created for bone analysis within the region of the osteotomy. Within the area surrounding the osteotomy, a cuboidally shaped VOI (A-VOI) measuring 0.4 × 0.16 × 0.533 cm was extracted. A further VOI (B-VOI) was created to identify the borders of the newly formed bone and, if still recognizable, the osteotomy. The A-VOI was subtracted from the B-VOI, leaving a smaller VOI (C-VOI) representing a standardized area within the osteotomy (Figure 3).

### 2.7 Histological Preparation: Paraffin

For fixation, the samples were placed in 4% paraformaldehyde for 4 days. Subsequently, the paraformaldehyde was washed out, and the bones were placed in phosphate-buffered saline (Waldeck GmbH & Co.KG, Münster, Germany) at 4 °C until the μCT scans were performed. The samples were decalcified in EDTA solution (Carl Roth GmbH & Co.KG, Karlsruhe, Germany) for at least 6 weeks at 37 °C, dehydrated in a battery of ascending alcohol series, and infiltrated with paraffin using a tissue processor (Leica TP 1020, Leica Biosystems GmbH, Nussloch). After embedding the samples, sections of 4 μm thickness were conducted with a microtome (Leica Biosystems Nussloch GmbH, Nussloch, Germany). Following initial slide preparation according to Table 1, a Movat’s pentachrome staining and an immunohistochemical staining for alpha-smooth muscle actin were performed according to Tables 2 and 3, respectively, Pictures were digitized using a digital light microscope (Leica DM6B, Leica microsystems CMS, Wetzler, Germany) and a digital camera (Leica DMC 4500, Leica microsystems (Switzerland) Ltd., Heerbrugg, Switzerland) with the LAS X software (Leica Application Suite X, Version: 3.7.5.24914). Automatic mosaic stitching was performed by using the systems software.

**Table 1:** Slide Preparation before Staining^a^

step	agent	time
deparaffinization	xylene	2 × 10 min
rehydration in descending alcohol solution	2 × 100% ethanol, 96% ethanol, 80% ethanol, 70% ethanol, aqua destillata	2 min each

a Xylene (Fisher Scientific, Loughborough, UK), ethanol (Carl Roth GmbH & Co.KG, Karlsruhe, Germany).

**Table 2:** Protocol of Movat’s Pentachrome Staining^a^

step/agent	time
3% acidic acid	3 min
1% Alcian blue in 3% acidic acid	30 min
3% acidic acid	4 min
aqua destillata	10 s
alkaline ethanol (10:1 96% ethanol/ammonia)	60 min
main water	10 min
aqua destillata	10 s
brilliant crocein acid fuchsine	15 min
0.5% acidic acid	10 s
5% phosphotungstic acid	20 min
0.5% acidic acid	1 min
100% ethanol	3 × 2 min
Saffron du Gatinais	60 min
xylene	2 × 2 min
mounting with Vitroclud	

a Acidic acid (Merck KGaA, Darmstadt, Germany), Alcian blue (Sigma-Aldrich Chemie GmbH, Steinheim, Germany), ethanol (Carl Roth GmbH & Co.KG, Karlsruhe, Germany), ammonia (Merck KGaA, Darmstadt, Germany), brilliant crocein acid fuchsine (Waldeck GmbH and Co. KG, Münster, Germany), phosphotungstic acid (Waldeck GmbH and Co. KG, Münster, Germany), Saffron du Gatinais (Waldeck GmbH and Co. KG, Münster, Germany), xylene (Fisher Scientific, Loughborough, UK), and vitroclud (R.Langenbrinck GmbH, Emmendingen, Germany).

**Table 3:** Protocol of Alpha-SMA Staining^a^

step	agent	time
washing	PBS	2 × 3 min
blocking	PBS/2% BSA/5% normal serum horse	30 min
1st antibody	mouse antihuman smooth muscle actin in DAKO diluent	60 min
washing	PBS	2 × 3 min
	vector alkaline phosphatase	30 min
washing	PBS	2 × 3 min
preincubation	chromogen buffer pH 8.2	2 × 3 min
	vector substrate	5 min under microscopic control
interruption	PBS	2 × 3 min
counterstaining	Mayer’s hemetoxylin	2 min
bluing	main water	5 min
	aqua destillata	10 s
mounting	Aquatex	

a Phosphate-buffered saline (PBS) (Waldeck GmbH & Co.KG, Münster, Germany), bovine serum albumin (BSA) (fraction V, igG free, NZ origin, C. Roth GmbH, Karlsruhe, Germany), normal serum (Vector Laboratories, Inc., USA, Newark USA), first antibody (Monoclonal Mouse Anti-Human-SMA #Clone 1 A4 (1200), Dako diluent), Dako diluent (Dako Antibody diluent with background-reducing components, Agilent Technologies Denmark ApS, Glostrup, Denmark), second antibody (horse antimouse, rat absorbed, biotinylated, Vector Laboratories, Inc., Burlingame, CA, USA), alkaline phosphatase (ImmPact Vector Red Substrate Kit, alkaline phosphatase, Vector Laboratories, Inc., USA, Newark USA), chromogen buffer made from tris-hydrochloride (Roche Diagnostics Deutschland GmbH, Mannheim, Germany), tris-base (Roche Diagnostics Deutschland GmbH, Mannheim, Germany), sodium chloride (Merck KGaA, Darmstadt, Germany), Mayer’s hematoxylin (Merck KGaA, Darmstadt, Germany), and aquatex (Merck KGaA, Darmstadt, Germany).

### 2.8 Histological Preparation: PMMA

During all of the following processes, the samples were protected from UV radiation to prevent fading of the fluorescence. For fixation, the samples were placed in 10% formaldehyde for 7 days. Subsequently, the formaldehyde was washed out, and the samples were dehydrated in an ascending alcohol series in accordance with Table 4. The samples were then placed in xylene (Fisher Scientific, Loughborough, UK) for 24 h and embedded in Technovit 9100 New (Heraeus Kulzer, Hanau, Germany). Each hardened block was ground to the central level of the screws, and nondecalcified sections were prepared with a thickness of approximately 100 μm according to Rendenbach et al. (2021).^38^ After acquiring the pictures for the fluorescent activity, staining was performed in Giemsa in accordance with Table 5, and the pictures were digitized using a digital light microscope with the AxioVision software (Axio Cam MRc5, Carl Zeiss Mikroskopie, Jena, Germany). Manual stitching was performed using the system's software.

**Table 4:** Protocol of Dehydration of PMMA Samples^a^

**agent**	**time**
70% ethanol	7 days
80% ethanol	7 days
96% ethanol	2 × 7 days
100% ethanol	2 × 7 days

a Ethanol (Carl Roth GmbH & Co.KG, Karlsruhe, Germany).

**Table 5:** Protocol of Giemsa Staining^a^

agent	time
1% formic acid	1 min
watering with main water	5 min
aqua destillata	2 min
Giemsa azur-eosin-methylene blue	15 min
100% ethanol	rinsing
Giemsa azur-eosin-methylene blue	20 min
100% ethanol	1 min
100% ethanol	30 s
xylene	1 min
xylene	1 min

a Formic acid (Merck KGaA, Darmstadt, Germany), ethanol (Carl Roth GmbH & Co.KG, Karlsruhe, Germany), Giemsa azur-eosin-methylene blue (Merck KGaA, Darmstadt, Germany), and xylene (Fisher Scientific, Loughborough, UK).

### 2.9 Sequential Polychrome Labeling

To visualize osteogenic activity, the fluorescent signal of the polychrome labeling was detected by using a digital fluorescence microscope (Keyence Corporation, BZ-X810, All-in-One, Osaka, Japan). Automatic mosaic stitching was performed using the system's software.

Three fluorescent substances were visualized using different filters: Cy5 (OP-87766, Keyence Corporation, Osaka, Japan Firma) for alizarin, GFP (OP-87763, Keyence Corporation, Osaka, Japan Firma) for calcein, and the combination of Cy5 and TRITC (OP-87764, Keyence Corporation, Osaka, Japan Firma) for xylenol. Following digitization, the pictures were imported to ImageJ 1.53o (National Institutes of Health, Rockville, USA), and a region of interest (ROI) of 5 mm width was set to analyze the amount of fluorescent activity within and surrounding the osteotomy for a quantitative analysis. The height of the ROI was adapted to the depth of the osteotomy. Using the HSB (high saturation brightness) color space, each of the three different fluorescent colors was segmented and extracted from the picture for each sample individually. These segmented pictures were then transformed into black and white pictures, with black representing the former fluorescent color. The number of black pixels was counted automatically.

### 2.10 Histomorphometric Analysis

To evaluate the tissue quality within the osteotomy region and to evaluate void formation close to the implant side, we analyzed mineralized bone (MinB), cortical bone (CoB), connective tissue (ConT), and void area (VoA) using a threshold-based segmentation in Movat’s pentachrome and Giemsa-stained samples and applying a self-implemented macro for histomorphometric tissue quantification. We performed a manual adjustment where necessary to ensure the precise capture of the tissue. The segmented tissue was then quantified within the regions of interest (ROIs).

For Movat’s pentachrome, we created an ROI representing the originally set osteotomy.

To investigate the bone-implant contact (BIC) in Giemsa-stained samples, we created an ROI including the inner borders of the two screws left and right to the osteotomy (ROI ScL and ScR) as well as an ROI including the miniplate (ROI Pl). ROI Pl had specific parameters. The height results from the plate height and an additional 0.5 mm. For magnesium-based miniplates, the height results in 2.25 mm; and for titanium-based miniplates, 1.5 mm. The width of the ROI Pl was set at 7 mm.

An immunohistochemical staining for alpha-SMA was performed. The number of vessels was calculated using ImageJ and a self-implemented macro. Two regions of interest were investigated: one representing the area between and surrounding the two inner screws (ROI A) and a second representing the osteotomy area (ROI-O). Results are reported as vessel count (Vc) over the total ROI-area.

### 2.11 Statistical Analysis

Data collected with ImageJ were automatically saved as a text file and then transformed into Microsoft Excel (Version 16.0.5404.1000, Microsoft Corporation, Redmond, WA, USA). Following the exclusion of normal distribution, a Mann–Whitney *U* test was performed to compare the groups. Statistical significance was defined as *p* < 0.05. Results are reported as mean values ± standard error of the mean (SEM). Graphs were created using GraphPad Prism, Version 9.5.0 (GraphPad Software LLC, San Diego, CA, USA), demonstrating minimal and maximal values as whiskers and the median plotted as the horizontal line within the box. Significant results are shown by asterisks. One asterisk represents a *p* value ≤ 0.05, and two asterisks represent a *p* value ≤ 0.001.

### 2.12 Blinding vs Nonblinding

Investigators could not be blinded throughout the surgical procedure, tissue preparation, and data acquisition due to differences in the implant’s color and diameter as well as the necessity of removing titanium plates for μCT analysis and paraffin embedding.

## 3 Results

Postoperative hematoma within the surgical area occurred in 11 out of 24 animals. Nine of these were not palpable anymore on the fourth postoperative day, whereas one hematoma persisted until the 18th postoperative day. After finalization, micro-computed tomography was used to detect plate fractures in 11 out of 12 plates within the magnesium group. All fractures were in the two-screw holes adjacent to the osteotomy. One plate fracture was recorded in the titanium group. Radiological observation during the healing period did not reveal severe dislocation of these fractured plates, and the animal’s health or food intake was at no time jeopardized during the trial. No gas formation was noted in the soft tissue. No wound-healing disorders were observed.

### 3.1 Micro-computed Tomography Analysis

**Figure 4:**
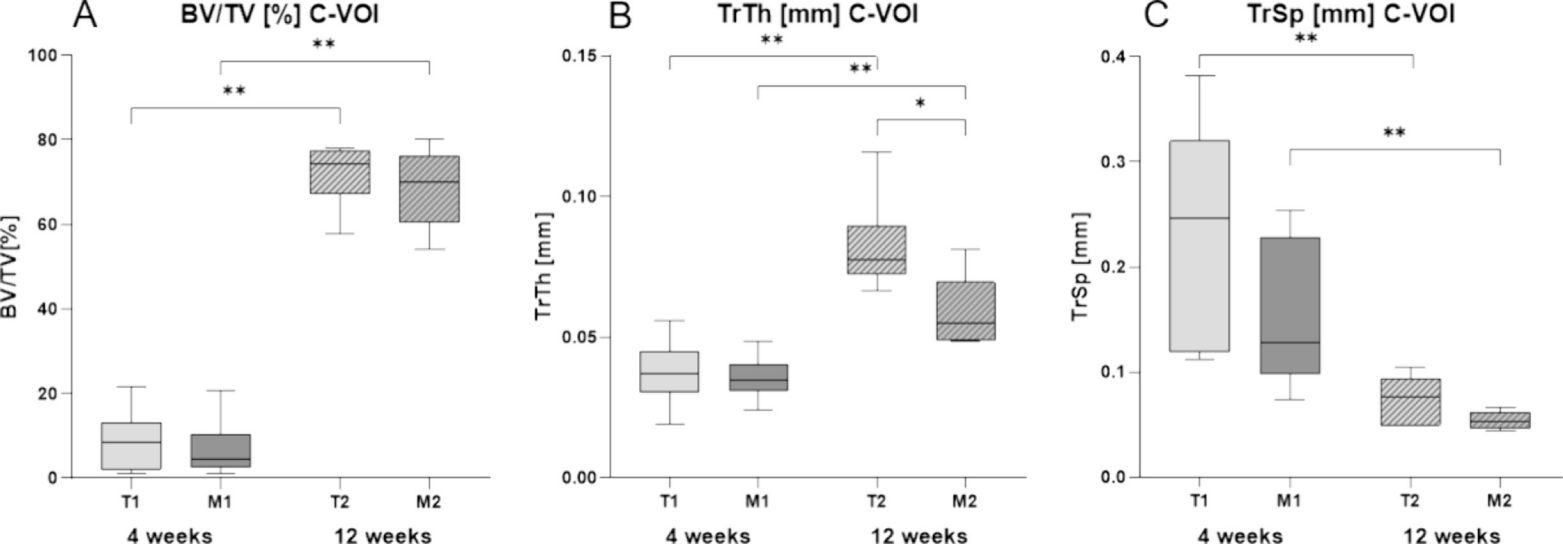
Results of micro-computed tomography analysis within the VOI area representing the inner part of the osteotomy. (A) Quantification of bone tissue demonstrated similar bone quantity in the magnesium and titanium groups after 4 and 12 weeks of healing process. (B) Quantification of the trabecular thickness showed significantly thinner trabeculae after 12 weeks in the magnesium group. In both groups, significant thickening of the trabeculae was observed over time. (C) Quantification of trabecular separation shows no significant difference in the development between magnesium and titanium.

Using 3D μCT analysis, the bone tissue was quantified as a percentage of the total area. This parameter is further termed BV/TV (bone volume/total volume). Morphological analyses were conducted using the average trabecular thickness (TrTh) and average trabecular separation (TrSp) in millimeters (Figure 4).

BV/TV analysis of the earlier bone healing stages after 4 weeks demonstrated a slightly lower percentage of bone volume in the magnesium group with no significant difference (BV/TV: T1: 8.74 ± 2.30%, M1: 6.83 ± 2.89%, *p* = 0.589). After 12 weeks, this slight difference persisted (BV/TV: T2: 71.99 ± 3.13%, M2: 68.58 ± 3.74%, *p* = 0.394). These results reveal that the bone volume within the fracture site is not significantly disturbed in the early and late healing stages by the presence of magnesium compared to titanium. The calculations demonstrated a markedly higher percentage of bone volume after 12 weeks in both groups, representing the healing progression (BV/TV: T1: 8.74 ± 2.30%, T2: 72.00 ± 3.13%, *p* = 0.00022 and M1: 6.83 ± 2.89%, M2: 68.58 ± 3.74%, *p* = 0.00022). The results in this model suggest that, after 4 weeks, the thickness of trabecular structures did not differ between titanium and magnesium groups (TrTh: T1: 0.04 ± 0.005 mm, M1: 0.04 ± 0.003 mm, *p* = 0.699). After 12 weeks, trabeculae formed in the magnesium group were significantly thinner than in the titanium group (TrTh: T2: 0.08 ± 0.01 mm, M2: 0.06 ± 0.01 mm, *p* = 0.04). In both groups, the trabecular thickness increased significantly over the healing time (TrTh: T1: 0.04 ± 0.01 mm, T2: 0.08 ± 0.01 mm, *p* = 0.002 and M1: 0.04 ± 0.003 mm, M2: 0.06 ± 0.01 mm, *p* = 0.004).

There was no difference regarding the trabecular separation in earlier or later healing stages (TrSp: T1: 0.23 ± 0.04 mm, M1: 0.15 ± 0.03 mm, *p* = 0.13 and T2: 0.07 ± 0.01 mm, M2:0.05 ± 0.003 mm, *p* = 0.132).

### 3.2 Qualitative Evaluation of Tissue Histology

**Figure 5:**
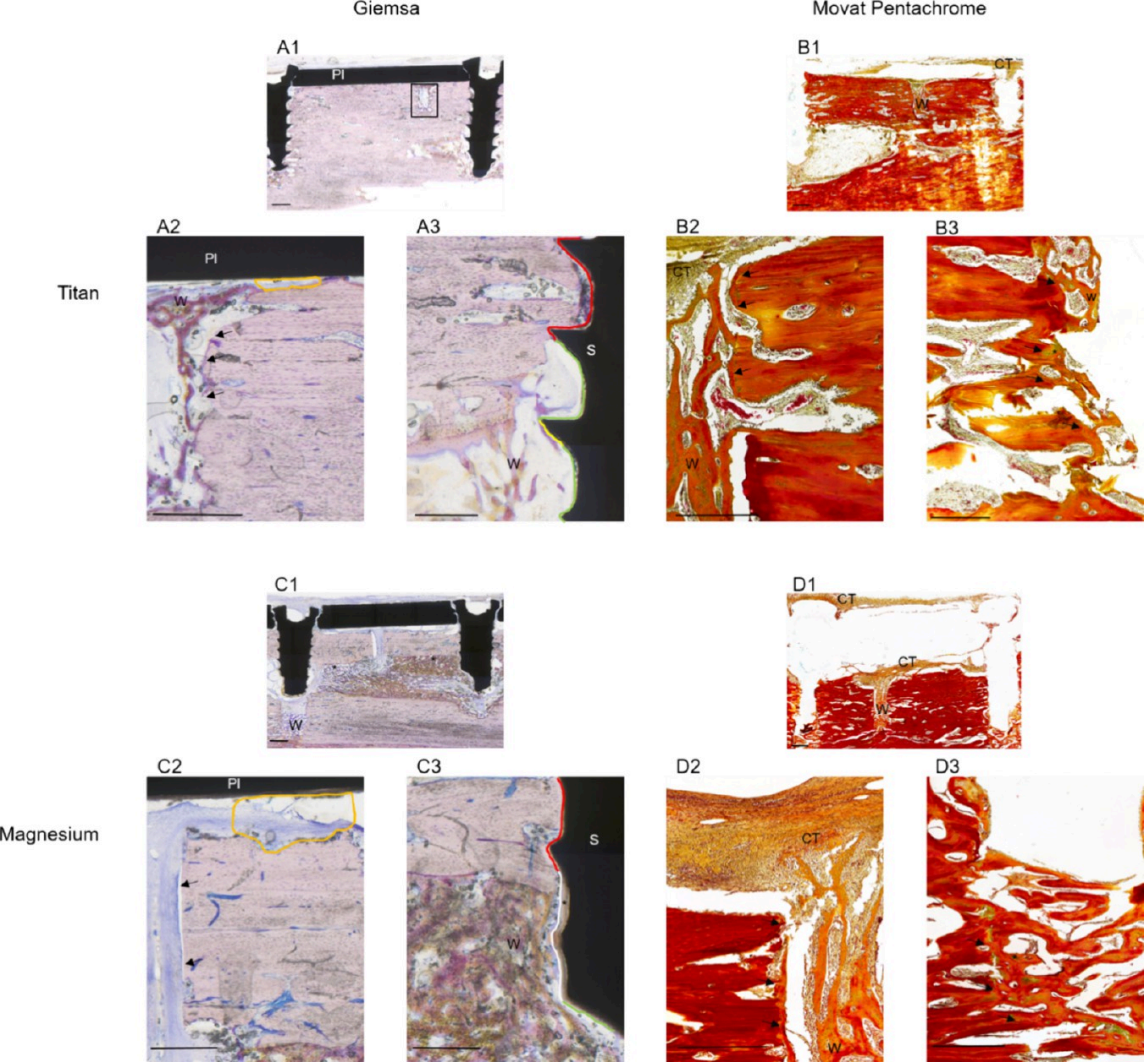
Histology of tissue reaction after 4 weeks. Legend: Pl = miniplate, S = screw, Ct = connective tissue, W = woven bone. (A) Titanium sample, stained with Giemsa. (A1) Overview. Miniplate aligns with bone border. Bone union within the osteotomy (rectangle) did not occur at this point. Scale bar: 1 mm. (A2) Magnification of osteotomy, surrounding cortical area, and plate. Woven bone within osteotomy. Arrows indicate the border of cortical bone. No strong periosteal reaction can be identified. A small fringe of the void area is visible between the periosteum and the plate, indicated by the orange border. Scale bar: 0.5 mm (A3) Magnification of the right screw. Screw material is in contact with different tissue types indicated by different colors (red = compact bone, green = void area, yellow = woven bone). At the lower part, endosteal reaction with woven bone formation is visible. Scale bar: 0.5 mm. (B) Titanium sample, 4 weeks, stained with Movat’s pentachrome. (B1) Overview: connective tissue above the plate. Woven bone formation occurs especially within the lower part of the osteotomy. Scale bar: 1 mm. (B2) Magnification of osteotomy and surrounding cortical area; clearly visible border between lamellar, cortical bone, and woven bone within the osteotomy indicated by arrows. In the upper part, there was mostly unmineralized connective tissue. Scale bar: 0.5 mm. (B3) Magnification of screw area: Screw is removed due to sample preparation. Woven bone formation is adjacent to screw void. The differentiation between woven and lamellar bone is not as clear as that in the osteotomy area, indicated by arrows. Islands of green staining (star symbol) represent the presence of cartilage formation, indicating endochondral ossification. Scale bar: 0.5 mm. (C) Magnesium sample, 4 weeks, stained with Giemsa. (C1) Overview: Endosteal bone formation is visible, indicated by star symbols. Woven bone formation within the predrilled screw hole is visible. No bone union occurred at this point. Scale bar: 1 mm. (C2) Magnification of osteotomy area: Effects of plate degradation are visible as more void area, and connective tissue is visible between the cortical bone and miniplate, indicated by orange borders. Arrows indicate the cortical bone borders. Scale bar: 0.5 mm. (C3) Magnification of right screw: Corrosion material is visible and indicated with a star symbol. Screw material is in contact with different tissue types indicated by different colors (red = cortical bone, white = connective tissue, and green = void area). Scale bar: 0.5 mm. (D) Magnesium, 4 weeks, stained with Movat’s pentachrome. (D1) Overview: In this staining, connective tissue formation is visible above and below the former plate. Scale bar: 1 mm. (D2) Magnification of the osteotomy area: Woven bone formation is visible in the lower and middle parts of the osteotomy. In the upper part, mostly unmineralized connective tissue is visible. Borders of cortical bone are clearly visible and indicated with arrows. Overall, a similar tissue composition is visible as in the titanium sample. Scale bar: 0.5 mm. (D3) Magnification of right screw area: The former drilling hole is filled with new, woven bone. Borders between cortical bone and woven bone are indicated by arrows. Islands of green staining (star symbol) represent the presence of cartilage formation indicating endochondral ossification. Scale bar: 0.5 mm.

No full osteotomy union was observed after 4 weeks. Bone formation within the osteotomy was detected in three magnesium and four titanium samples. In all samples, connective tissue was observed within and surrounding the osteotomy area. In two magnesium samples, clear, tissue-free, round structures representing the degradation process were identified surrounding the osteotomy. Periosteal and endosteal bone formation was found in all samples (Figure 5).

**Figure 6:**
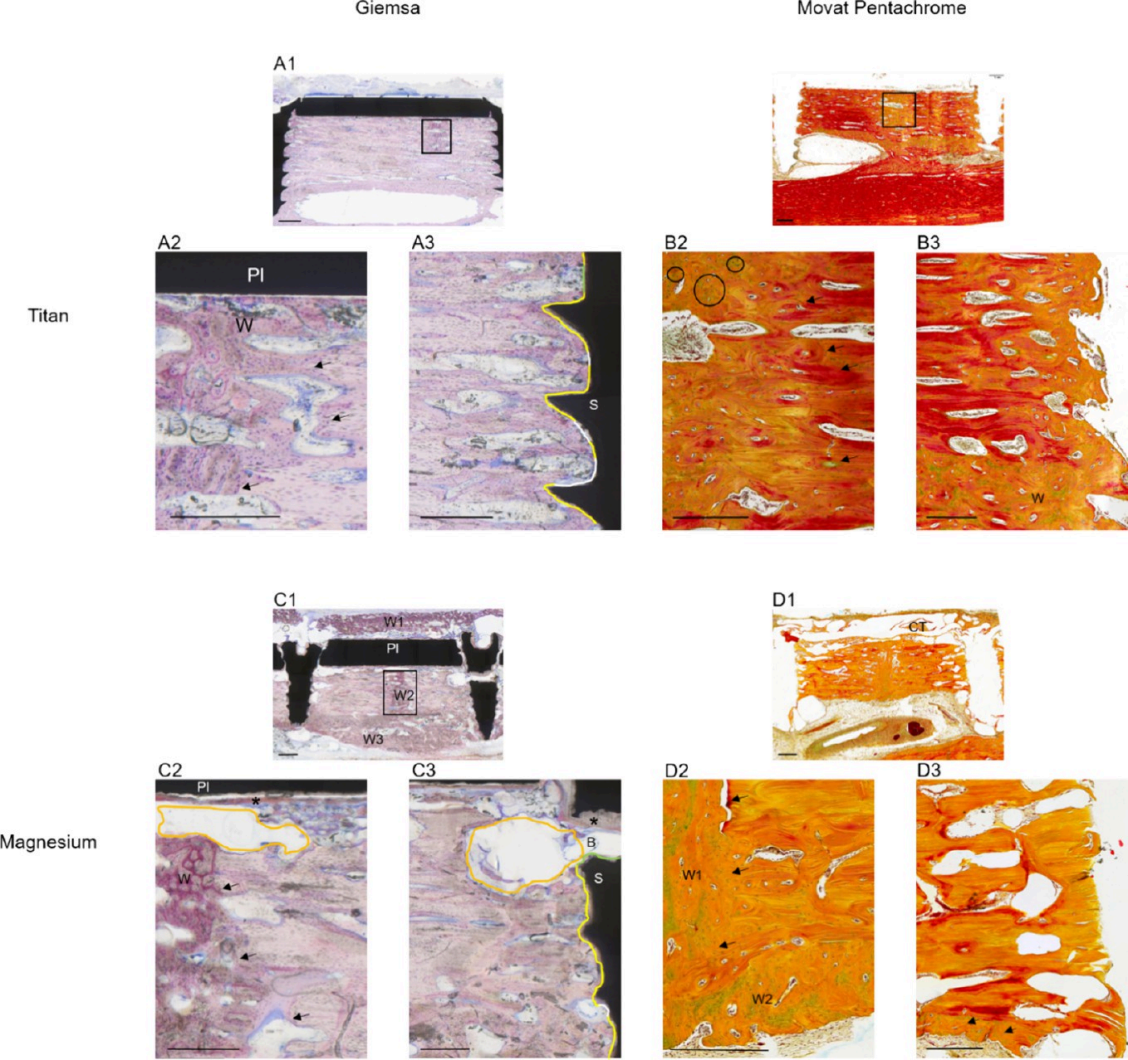
Histology of tissue reaction after 12 weeks. Legend: Legend: Pl = miniplate, S = screw, Ct = connective tissue, W = woven bone. (A) Titanium, stained with Giemsa. (A1) Overview: After 12 weeks, full bridging of the osteotomy took place (black rectangle). Scale bar: 1 mm. (A2) Magnification of the osteotomy area; plate in contact with mostly newly formed bone. Borders of cortical bone are indicated with arrows. Scale bar: 0.5 mm. (A3) Screw material is in contact with mostly newly formed woven bone (yellow border). White border and green border represent connective tissue and void area, respectively. Scale bar: 0.5 mm. (B) Titanium sample, 4 weeks, stained with Movat’s Pentachrome. (B1) Overview: Complete bone union occurred (black rectangle). Scale bar: 1 mm. (B2) Magnification of the osteotomy area. Circled areas indicate green stained cartilage tissue indicating endochondral ossification. Differentiation between lamellar and new bone is indicated by arrows. Scale: 0.5 mm. (B3) Magnification of right screw; mostly woven bone surrounding the screw. Bone material seems to be denser than after 4 weeks. Scale bar: 0.5 mm. (C) Magnesium, 12 weeks, stained with Giemsa. (C1) Overview: bone formation above the plate, within the osteotomy, and endosteally (indicated by W1, W2, and W3, respectively). Full bone union took place, indicated by a black rectangle. Screw breakage occurred on the right side. Scale bar: 1 mm. (C2) Magnification of osteotomy area; new, woven bone within osteotomy area. Borders of cortical bone indicated with arrows. Corrosion material of the miniplate is indicated with a star symbol. Void area visible underneath the plate is indicated by orange border. Scale bar: 0.5 mm. (C3) Magnification of right screw: Screw material is in contact with different tissue types represented by different border colors (woven bone = yellow, white border = connective tissue, green border = void area). At the point of screw breakage (B), more void area exists (orange border). This may be due to higher degradation speed, as more magnesium surface is in contact with the physiological area. Scale bar: 0.5 mm. (D) Magnesium, 12 weeks, stained with Movat’s Pentachrome. (D1) Overview: In the area of plate degradation, the formation of connective tissue can be observed. Scale bar: 1 mm. (D2) Magnification of osteotomy area; woven bone within osteotomy area (W1) and endosteally (W2). Arrows indicate the border between cortical bone and new bone. Bone density seems to be higher than after 4 weeks. Scale bar: 0.5 mm. (D3) Magnification of screw area: Differentiation between new bone and cortical area is not clearly visible. Endosteal bone formation took place, indicated by arrows. Scale bar: 0.5 mm.

After 12 weeks, complete bone union was observed in all samples in the magnesium and in five samples in the titanium group. In one titanium sample, only the upper part of the osteotomy united, and the lower part was mainly filled with connective tissue. Mostly woven bone was identified within the osteotomy area in both groups (Figure 6).

There was no difference in the amount of mineralized bone between the two groups after 4 weeks (MinB: T1: 26.16 ± 9.21%, M1:22.15 ± 7.99%, *p* value = 0.818). No significant difference was found regarding the connective tissue after 4 weeks. (ConT: T1: 59.89 ± 11.21%, M1: 50.42 ± 6.92%, *p* = 0.818). Significantly more void area was found in the magnesium group after 4 weeks (VoA: T1: 12.57 ± 3.18%, M1: 25.64 ± 4.10%, *p* = 0.041). The void area decreased significantly from 4 to 12 weeks in the magnesium group (VoA: M1: 25.64 ± 4.10%, M2: 10.09 ± 2.89%, *p* value = 0.026).

**Figure 7:**
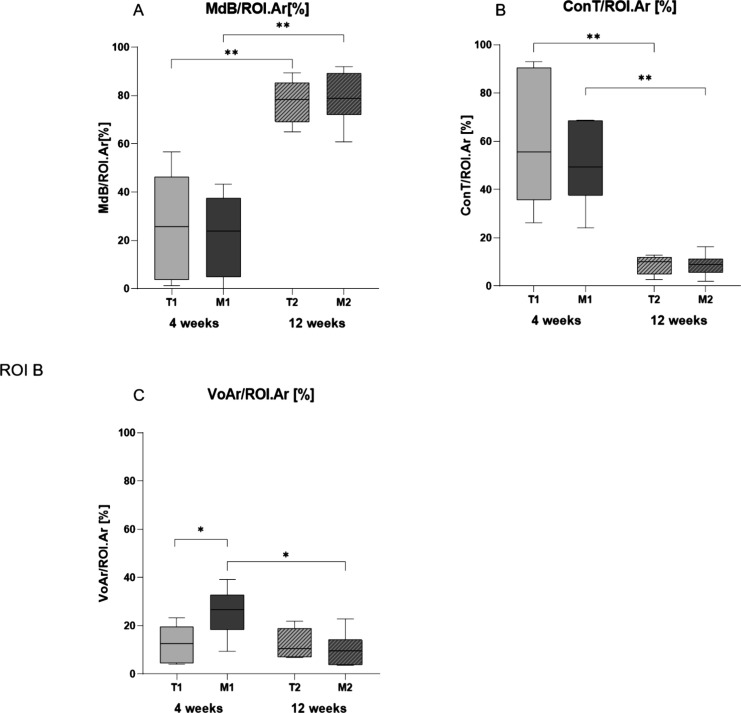
Results of tissue quantification within ROI representing the osteotomy area. (A) Quantification of the newly formed mineralized bone demonstrated no statistically significant differences comparing the magnesium and titanium groups, which indicated similar bone healing development. (B) Quantification of the connective tissue showed no significant differences when comparing the magnesium and titanium groups, indicating sufficient tissue development over time in both groups. (C) Quantification of the void area showed significantly more void area within the magnesium group, most likely owing to magnesium degradation. As the bone healing progresses, the void area decreases in both groups.

There was no difference in tissue composition following 12 weeks (MinB: T2: 77.56 ± 3.61%, M2: 79.06 ± 4.46%, *p* = 0.699; ConT: T2: 8.82 ± 1.60%, M2: 8.75 ± 1.91%, *p* = 0.818; and VoA: T2: 12.47 ± 2.52%, M2: 10.09 ± 2.89%, *p* = 0.589) (Figure 7).

In both groups, significantly more bone tissue and significantly less connective tissue occurred between 4 and 12 weeks, which indicate a progressing bone formation over time.

### 3.3 Histological Quantification of Tissue Quality Surrounding the Implant

**Figure 8:**
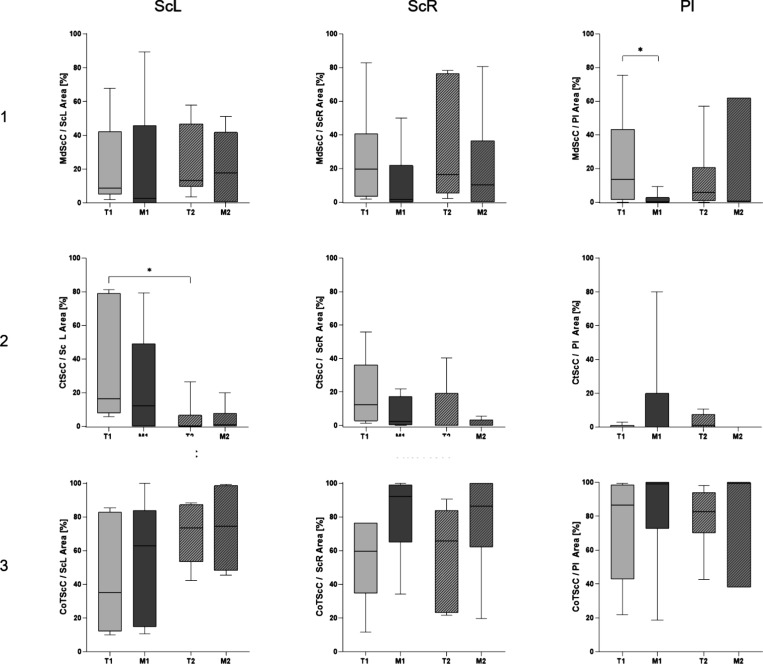
Quantification of tissue quality surrounding the implant material. Row 1: quantification of mineralized bone in contact with screw and plate material. After 4 weeks, significantly less mineralized bone is in contact with the implant in the magnesium group compared to the titanium group. Row 2: cortical bone in contact with implant material. Row 3: quantification of connective tissue in contact with screw and plate material.

The quantification of the tissues surrounding the miniplates and screws demonstrated no differences between the implant materials except for a higher percentage of BIC in the area below the miniplate (ROI Pl) in the titanium group (MdSc: T1: 22.74 ± 11.53%, M1: 1.62 ± 1.50%, *p* = 0.035; Figure 8). After 4 weeks, most of the newly formed bone in contact with the plate was located periosteal near the osteotomy, whereas after 12 weeks, the formation of new bone took place more disseminated throughout the length of the plate.

### 3.4 Vascularization

**Figure 9:**
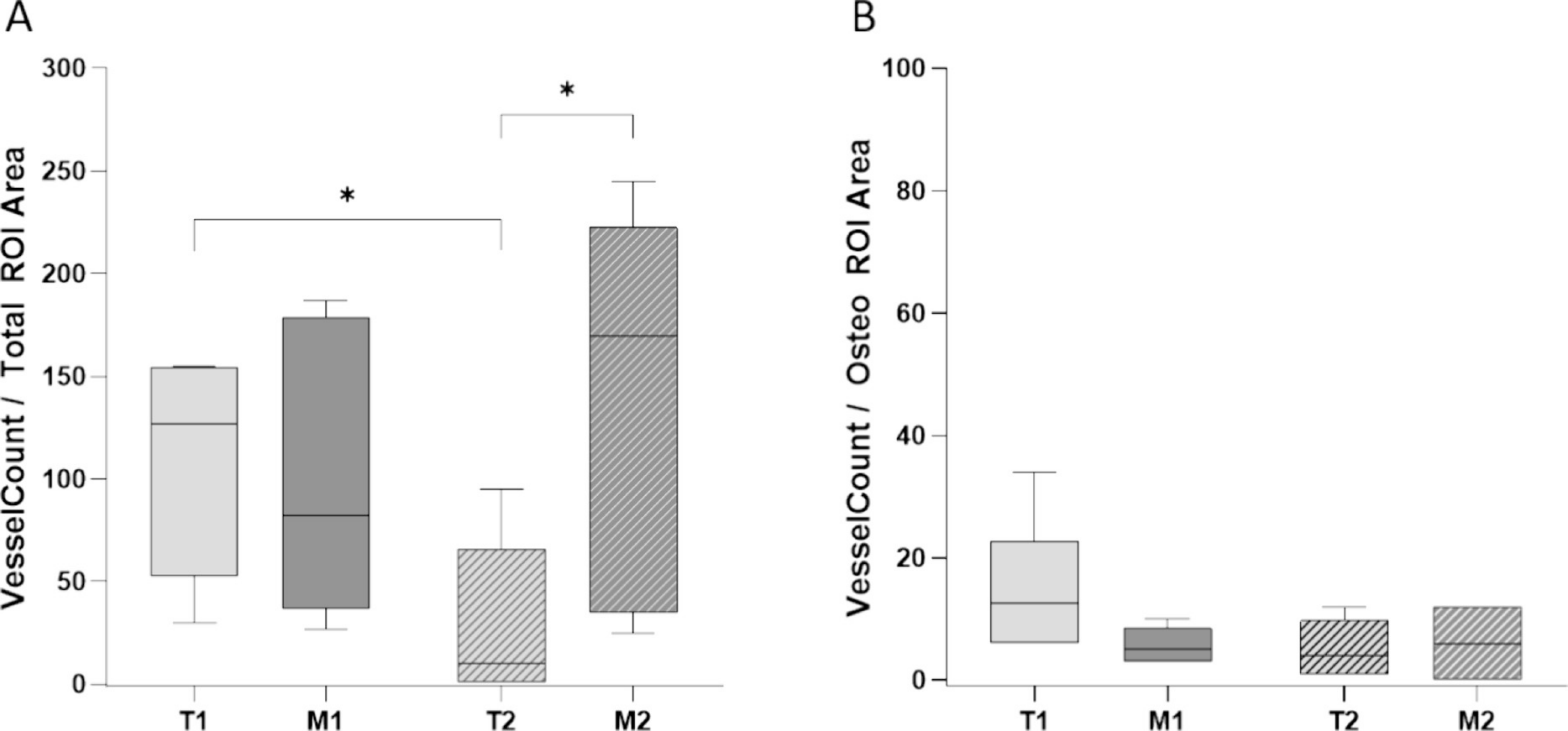
Graphical results of vessel quantification. (A) Vessel quantification was in the total ROI surrounding the two screws adjacent to the osteotomy. After 12 weeks, there were significantly more vessels in the magnesium group. This indicates a higher inflammatory response. Interestingly, in the titanium group, we observed considerably fewer vessels after 12 weeks compared to 4 weeks, whereas in the magnesium group, we observed a tendency for more vessels over time. This indicates that magnesium causes an additional inflammatory stimulus compared with the gold-standard therapy with titanium as an implant material. (B) Vessel quantification in the ROI is surrounding only the osteotomy area. A similar result in the development of vessels can be observed, as we see a decreasing tendency in the titanium group over time and an increasing tendency in the magnesium group. These results are not statistically significant.

Within ROI A, no statistically significant difference was identified regarding vessel count in the earlier healing stages (Vc: T1: 108.70 ± 22.21, M1: 99.00 ± 27.87, *p* = 0.9372). After 12 weeks, significantly more vessels were counted in the magnesium group (Vc: T2: 28.83 ± 15.85, M2: 143.70 ± 37.76, *p* = 0.024). The vessel counts between 4 and 12 weeks significantly decreased in the titanium group (Vc: T1: 108.70 ± 22.21, T2: 28.84 ± 15.85, *p* = 0.015). A nonsignificant increase was observed in the magnesium group (Vc: M1: 99.00 ± 27.87, M2: 143.70 ± 37.76, *p* = 0.4848) (Figure 9).

Within the osteotomy area (ROI O), no differences were observed regarding the vessel counts (Vc: T1: 15.00 ± 4.52, M1: 5.67 ± 1.17, *p* = 0.091; T2: 5.17 ± 1.83, M2: 6.00 ± 2.54, *p* = 0.916; T1: 15.00 ± 4.52, T2: 5.17 ± 1.83, *p* = 0.056; and M1: 5.67 ± 1.17, M2: 6.00 ± 2.54, *p* > 0.999). The number of vessels tended to decrease in the titanium group over the observation period, in contrast to the magnesium group.

### 3.5 Analysis of Polychrome Fluorescent Labeling

Polychrome sequential fluorescent labeling was performed to evaluate the osteogenic activity. Each injected fluorescent agent could be detected and analyzed. Osteogenic activity was quantified as fluorescent signals’ percentage of the ROI area. Following 7 days, the fluorescent signal (FluS) was predominantly noticeable in the periosteal and endosteal parts of the bone. Very little signal was identified within the osteotomy area. This pattern did not differ between the groups. After 21 days, more signal was visible throughout the sample than after 7 days postoperatively, and the calcein incorporation was equally distributed over the analyzed area. In comparison to the early alizarin signal after 7 days, more calcein activity was detected within the osteotomized area after 21 days. This increase in bone turnover was statistically significant and noticed in both the magnesium and the titanium groups (titanium: FluS day 7: 0.01 ± 0.003%, FluS day 21: 0.03 ± 0.01%, *p* = 0.03 and magnesium: FluS day 7: 0.01 ± 0.003%, FluS day 21: 0.06 ± 0.03%, *p* = 0.01).

After 12 weeks, we identified a more disseminated signal for all three fluorescent signals. In the magnesium group within the osteotomy, the signal appeared to be increased after 56 days compared to the signal after 35 and 77 days, indicating an elevated osteogenic activity at this point in time. The fluorescent signal differed markedly between days 56 and 77 (magnesium: FluS day 56: 0.09 ± 0.03%, FluS day 77: 0.03 ± 0.01%, *p* = 0.04).

**Figure 10:**
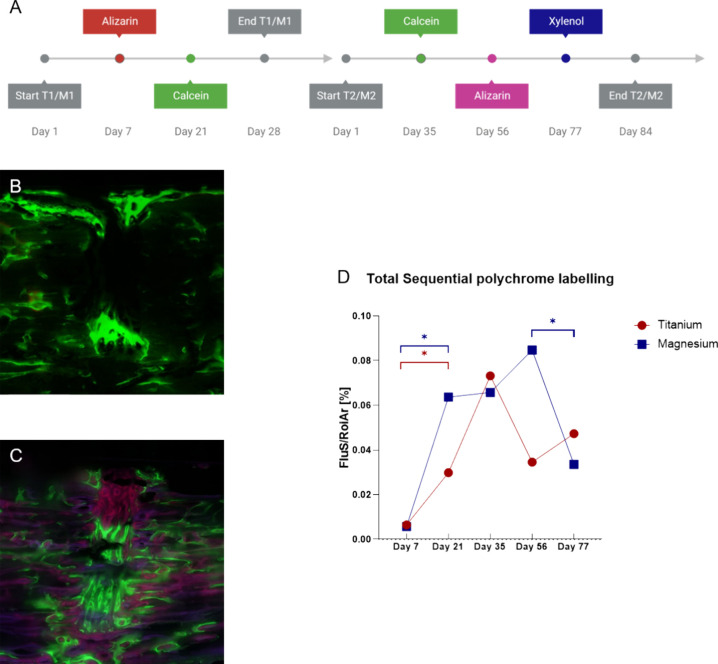
Polychrome fluorescent labeling. (A) Timeline of polychrome injections. Fluorescent signaling from calcein is visualized by green color, alizarin in the 4 week group by red color, in the 12 week group by pink color, and xylenol by blue color. (B) Magnification within the osteotomy area of a titanium sample after 4 weeks. Mostly fluorescent calcein signaling can be detected. Scale bar: 0.5 mm. (C) Magnification within the osteotomy area of a magnesium sample after 12 weeks. All three fluorescent signals can be detected. Scale bar: 0.5 mm. (D) Graphical presentation of fluorescent signaling of all injection points as a percentage of the total ROI area. No statistically significant differences were identified when comparing the two materials

In the titanium group, the highest osteogenic activity could be detected on day 35. This difference was not significant. Comparing the titanium and magnesium groups, we found no statistically relevant difference in the osteogenic activity after 7, 21, 35, 56, and 77 days (Figure 10).

## 4 Discussion

Various facets contribute toward the sufficiency of fracture healing ranging from influenceable aspects like fracture fixation and postoperative rehabilitation regime to uncontrollable factors such as fracture patterns, patient age, or concomitant diseases.^32,33^ To provide the best health care possible, modern research should focus on innovative strategies to lower patients’ health risks when intervention is needed. Among others, biomechanical aspects can determine the success of fracture healing as mechanical loads directly influence the cellular responses, leading to the regulation of bone metabolism, regeneration, and remodeling.^34^ This study aimed to investigate the potential efficacy and noninferiority of a magnesium-based human-standard-sized fracture fixation system at the mandible when compared to the gold standard with a titanium-based system.

Magnesium as a biodegradable material potentially reduces the risk for second interventions and complications like implant infection or extrusion when used as a fixating material.^2^ Besides decreasing patients’ health risk and pain, this aspect could reduce public health care expenses.^35,36^

Although sheep mandibles differ biomechanically and anatomically from their human equivalent, the dimensions are comparable, and the sheep mandibular osteotomy model represents rather a critical setting compared to humans because sheep do not unload the osteotomy fixation.^37,38^ Regarding the biomechanical differences between magnesium and titanium miniplates, several studies could show a comparable performance of the two materials. In silico, Orassi et al. (2022) developed a mandibular fracture model and predicted a similar biomechanical environment within the healing tissue at different fracture sides comparing magnesium and titanium miniplates. Fischer et al. (2022) demonstrated the biomechanical noninferiority of 1.5 and 1.75 mm WE43-MEO miniplates with PEO surface modification compared to 1.0 mm titanium miniplates in a sheep mandible osteotomy model ex vivo. To mitigate the risk of early plate failure in the context of the high mechanical load in the mandibular bone, this study was conducted with 1.75 mm WE43 MEO miniplates with PEO surface modification and 1.0 mm titanium miniplates. Previous research has demonstrated good biocompatibility of WE43 PEO surface modified in bone tissue.^39^ The application of thicker miniplates may entail a greater risk of adverse effects on the surrounding soft tissue and is accompanied by larger amounts of gas formation due to the higher amount of degrading magnesium. The effects of degrading magnesium are further discussed below. Yet, methods such as computer modeling facilitate the reduction of plate material within plates without forfeiting its benefits.^40^

Analyzing the degree of degradation of WE43 in comparison to WE43 with PEO surface modification, Rendenbach et al. (2021) showed conclusively that the degradation speed of WE43-PEO is significantly lower after 6 months with a residual screw volume of 62.9%.

The aim of the study was to understand the effects magnesium degradation may have on the bone healing process. Because of the extended grinding movement of the mandible, which occurs within the sheep natural behavior, the bone is prone to much higher mechanical stress compared to the human equivalent. For the reason of animal welfare, the study was conducted using a monocortical osteotomy within the diastema of the mandible, as human-standard size miniplates would not bear the higher mechanical stress. Even though the nonfractured lingual cortex was part of the load-sharing system, a load-sharing component was given to the fracture side as the failure of some plates indicates. A greater number of magnesium-based plates broke. Because of their elastic modulus, magnesium-based implants are more susceptible to deformation than titanium.^41^ Remarkably, neither broken titanium nor magnesium miniplates were dislocated, which indicate that the overall fixation was sufficient even with plate failure at specific screw holes. Severe interference with the fracture healing process was not detected as a good clinical outcome was observed in all groups with complete bone healing and no clinically visible functional disorder. Plate failure occurred in the two screw holes next to the osteotomy. This reflects the biomechanical studies of Fischer et al. (2022). However, to fully understand the mechanical behavior of degrading magnesium plates at even more distant healing stages, a longer observation period would be necessary. The literature gives interesting insights on methods to improve the corrosion rate and biomechanical stability of magnesium-based implants. These include geometrical optimization, deposition of polymeric layers using the dip-coating process, galvanic anodization, hydrothermal surface modifying coatings, biocoatings, or adding further alloying elements.^42−44^

### 4.1 Tissue Quality and Bone Microstructure

Radiological analyses of the osteotomy area indicate that magnesium-based implants lower bone density, even though this study found no significant difference. The current results are consistent with findings of studies by Schaller et al., who evaluated WE43-based human-standard size osteosynthesis plate/screw systems in a rib model and a load-sharing maxillofacial environment in minipigs.^23,45^ Contrary to the reports of Zhao et al, this study reported a lower trabecular thickness in the presence of magnesium.^46^ High trabecular thickness is related to high bone quality and stability.^47^ This contrary finding could be explained by differences in magnesium implant sizes between the studies. On the other hand, the present study demonstrated an increase in the trabecular separation and bone density over time, indicating bone maturation and growth in both groups and representing elevation of bone quality over time.

In the histological analysis, evidence was found for bone formation and bone union in both groups. An adequate morphological development in tissue quality from connective tissue to mineralized bone occurred regardless of the implant material. This corroborates the radiological findings.

Gas formation occurs because of degrading magnesium.^2^ In vivo approaches indicate that the gas consists of different gases. Gases reported to be part of the composition are mostly CO, H_2_, O_2_, and N_2_, although the current literature is divided concerning the exact gas composition.^48,49^ According to Kim et al. (2018), no extended side effects of the gas formation were reported in a clinical observation of the degradation process. In the present study, at earlier healing stages, significantly more plate material was in contact with newly formed bone in the titanium group. As periosteal bone formation was observed in both groups; this difference could be explained by greater amounts of gas between the plate and the bone. Nevertheless, in later healing stages, bone formation surrounding the plate increased within the magnesium group. Wound healing and bone healing within the osteotomy did not seem to be impeded at any time. This result is in line with the current literature stating that gas formation is highest in earlier healing stages.

### 4.2 Osteogenic Activity and Vascularization

The relationship between released magnesium ions and the bone’s osteogenic potential has been widely investigated, conclusively showing that magnesium enhances the osteogenic activity in vivo and in vitro.^50−52^ Among others, a positive effect of magnesium on subperiosteal bone formation was reported.^50^

Analyzing the osteotomy side, our findings did not indicate enhanced osteogenic potential in magnesium-fixated compared with titanium-fixated mandibular osteotomies. This result is consistent with the radiological and histological analyses of this study.

Nevertheless, we identified differences regarding the healing process between the two implant materials. Within the magnesium group, the highest osteogenic activity occurred after 56 days. In the titanium group, the highest activity was visible after 35 days. Although this difference was not significant in the titanium group, it may indicate that bone remodeling started earlier within the titanium group. This could correlate with the higher bone density and mineralization compared to magnesium at 4 weeks found in the present study. During magnesium degradation, magnesium ions become vacant and act as an additional stimulus for bone remodeling.^2^ This could explain the higher osteogenic activity in later healing stages within the magnesium group.

Higher vascularization results from a greater inflammatory response and consequently higher expression of angiogenic factors. This process is crucial to the bone healing process.^53^ Previous studies demonstrated an enhancing effect of magnesium ions on angiogenic factors.^54^ The calculations of the present study show that magnesium did not markedly elevate the revascularization after 4 weeks. In the load-sharing model, this effect seems to be equalized by the mechanical stimuli in both the magnesium and titanium group. However, at later bone healing stages, magnesium seems to have a significant influence that enhances the vascularization. This difference can be explained by the fact that, in the titanium group, no additional stimulus on the bone remodeling process occurred. Within the magnesium group, however, this stimulus is rooted from the ongoing degradation process of the implant material, resulting in higher vascularization.

In conclusion, the degradation of magnesium seems to have a positive impact on the osteogenic potential of the healing bone in later healing stages. This aspect appears to correlate with a higher inflammatory response. In the present study, this higher inflammatory activity did not seem to interfere with the stability of the fracture site or with the quality of the healing bone. A longer observation period could afford an insight into whether the degrading implant causes adverse effects in the late remodeling stages.

### 4.3 Tissue Reaction to Screw and Plate Failure

**Figure 11:**
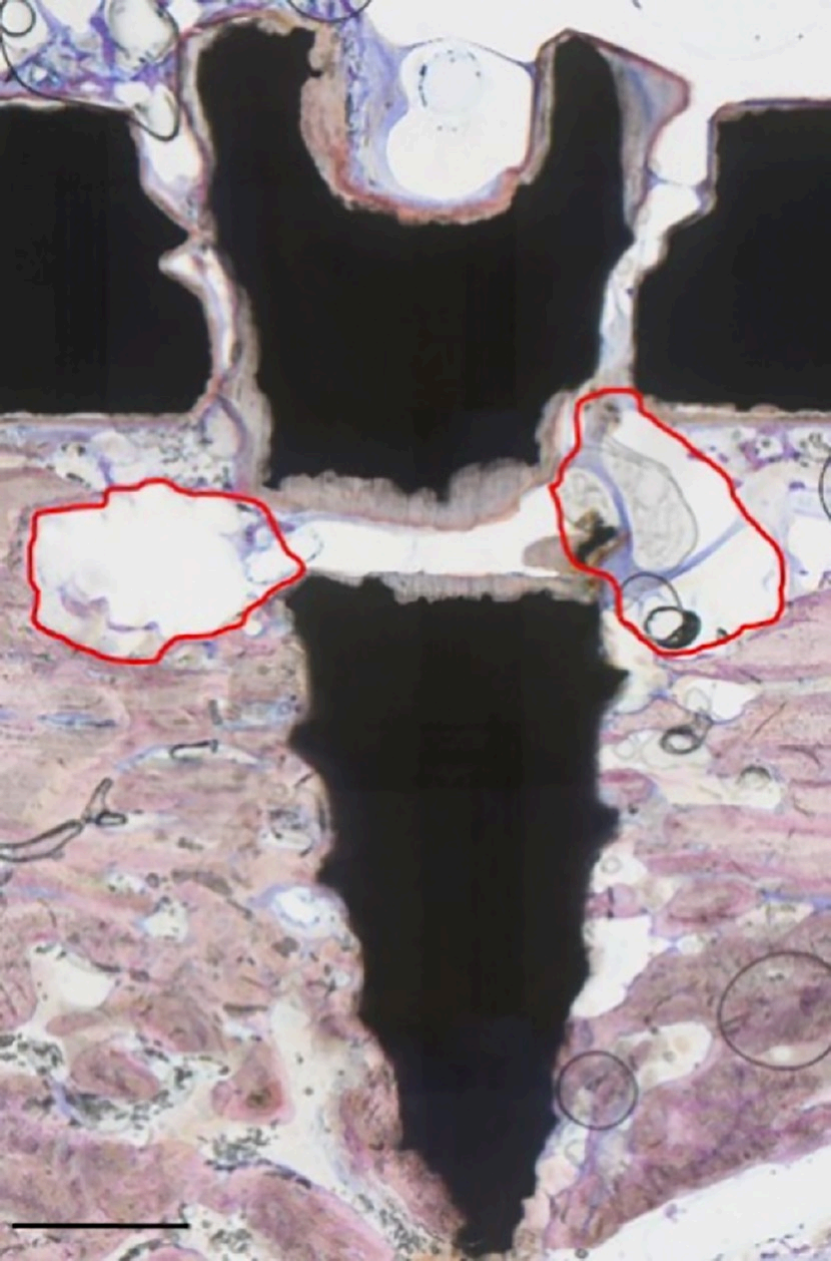
Tissue reaction to screw failure. Broken screw with adjacent gas formation is indicated by a red border. Scale bar: 1 mm.

An interesting phenomenon within the magnesium group was the tissue reaction adjacent to broken screws and plates. The degradation speed seemed to be accelerated at this point, and greater gas formation was visible in the surrounding area (Figure 11). This could be due to the interrupted degradation deceleration achieved by PEO surface modification when the implant surface is damaged and discontinued. We can conclude that as soon as material failure occurs, the magnesium alloy degrades at its natural speed. Levorova et al. reported that in nonmodified WE43-based screws in a rabbit tibia, the degradation speed accelerated because of degradation products after 12 and 16 weeks.^20^ In PEO-modified implants, this phenomenon will most likely occur regardless of screw or plate failure owing to the degradation of the PEO surface modification. This is not necessarily a negative aspect of magnesium-based implants, as the present study showed adequate bone healing irrespective of the implant material. On the contrary, as an additional stimulus, the degrading magnesium could elevate the quality of the remodeling process. Future research should thus focus on a complete understanding of the influence of degradation at distant healing stages, and this phenomenon should be considered when adequate usage of magnesium implants is to be achieved in more advanced fracture healing systems.

### 4.4 Conclusions

WE43 is already in clinical use for orthopedic and maxillofacial interventions.^25−29^ The implant sizes in clinical use have smaller dimensions. This study demonstrated that the application of human-standard size miniplates in a load-sharing fracture fixation is possible, as adequate bone healing with a good clinical outcome was visible. Nevertheless, changes were identified in the bone architecture of the newly formed bone and adjacent tissues, as well as the healing process. Eventually, longer observational periods, e.g., the complete resolution of the magnesium implants or complete remodeling in titanium plate fixation, should be considered prior to drawing final conclusions.

## Data Availability

The raw/processed data required to reproduce these findings can be shared upon reasonable request.
